# Clinical Outcomes of Polymer-Free Versus Polymer-Coated Drug-Eluting Stents in Patients With Coronary Artery Disease: A Systematic Review and Meta-Analysis

**DOI:** 10.7759/cureus.38215

**Published:** 2023-04-27

**Authors:** Mahima Khatri, Satesh Kumar, Kamran Mahfooz, FNU Sugandh, Deepak Dembra, FNU Mehak, Gianeshwaree Alias Rachna Panjwani, Hamza Islam, Rabia Islam, Syed Muhammad Ibn e Ali Jaffari, Tirath Patel, Ajay Kumar, Nomesh Kumar, Giustino Varrassi

**Affiliations:** 1 Medicine and Surgery, Dow University of Health Sciences, Karachi, PAK; 2 Medicine and Surgery, Shaheed Mohtarma Benazir Bhutto Medical College, Karachi, PAK; 3 Internal Medicine, New York Health and Hospital Corporation, Lincoln Medical Center, New York, USA; 4 Medicine, Ghulam Muhammad Mahar Medical College, Sukkur, PAK; 5 Medicine, Civil Hospital Karachi, Sukkur, PAK; 6 Surgery, Ghulam Muhammad Mahar Medical College, Sukkur, PAK; 7 Medicine and Surgery, Ghulam Muhammad Mahar Medical College, Sukkur, PAK; 8 Research, Punjab Medical College, Faisalabad, PAK; 9 Research, Faisalabad Medical University, Faisalabad, PAK; 10 Medicine and Surgery, Shalamar Medical and Dental College, Lahore, PAK; 11 Surgery, American University of Antigua, St. John, ATG; 12 Internal Medicine, MedStar Union Memorial Hospital, Baltimore, USA; 13 Surgery, Detroit Medical Center-Wayne State University of Sinai Grace, Michigan, USA; 14 Pain Medicine, Paolo Procacci Foundation, Rome, ITA

**Keywords:** systematic review, meta-analysis, cad, coronary artery disease, pc-des, pf-des, polymer coated, polymer-free, drug-eluting stent

## Abstract

Drug-eluting stents have transformed the treatment of coronary artery disease (CAD), and there are two types: polymer-free and polymer-coated stents. Polymer-free stents have a coating that is quickly absorbed by the body, whereas polymer-coated stents have a coating that remains on the stent surface. This meta-analysis and systematic review aimed to compare the clinical outcomes of these two stent types in patients with coronary artery disease. The literature and abstracts from significant databases were reviewed to compare polymer-free drug-eluting stents (PF-DES) and polymer-coated drug-eluting stents (PC-DES) for the treatment of coronary artery disease (CAD). The primary efficacy endpoints of the study were all-cause mortality and deaths from cardiovascular and non-cardiovascular causes. Among the secondary outcomes were incidences of myocardial infarction (MI), target lesion revascularization (TLR), target vessel revascularization (TVR), stent thrombosis, stroke, and major adverse cardiovascular events (MACEs). In terms of the primary outcomes, the combined analysis revealed a marginally lower risk of all-cause mortality (relative risk, RR (95% CI) = 0.92 (0.85, 1.00), p = 0.05, I^2^ = 0%) with the use of PF-DES versus PC-DES. Nonetheless, there was no significant difference in cardiovascular mortality (RR (95% CI) = 0.97 (0.87, 1.08)) or non-cardiovascular mortality (RR (95% CI) = 0.87 (0.69, 1.10), p = 0.25, I^2^ = 9%) between the groups. Furthermore, univariate meta-regression revealed that male gender and prior myocardial infarction were independently associated with an increased risk of all-cause mortality and cardiovascular disease. According to the current meta-analysis, no statistically significant differences existed in PF-DES and PC-DES outcomes. More extensive research is needed to investigate these findings further and establish their validity.

## Introduction and background

Coronary artery disease (CAD), a condition characterized by the accumulation of lipids, calcium, and other compounds in the arteries, has emerged as the greatest danger to the public's well-being. The pathophysiology of CAD involves thrombus formation within the artery leading to vascular obstruction. According to a World Health Organization (WHO) report, more than 60 million potential life years are wasted globally in Europe due to vascular diseases [1]. New advances in coronary stent devices have boosted the prognosis for patients with coronary artery disease. The successive generations of equipment have marked significant advancements in the configuration, framework, and component materials of stents. There are three types of stents, which are categorized as bare metal stents (BMS), durable polymer drug-eluting stents (DP-DES), and polymer-free drug-eluting stents (PF-DES) [1,2]. In current history, researchers have focused on a new generation of drug-eluting stents (DES), including biodegradable polymer DES (BP-DES). This innovative stent platform employs a bioresorbable polymer coating that allows for the sustained release of an antiproliferative agent.

Several improvements in stent design have been introduced to reduce the risk of very delayed stent thrombosis (ST) associated with the current gold standard DP-DES [2,3]. The innovation of biocompatible polymers is an approach for mitigating this negative effect. The second is the creation of a biodegradable polymer that dissipates over time, leaving only BMS behind. Theoretically, BP-DES has the benefit of leaving only the BMS after complete drug elution and polymer degradation, which may reduce vascular inflammation and the risk of late stent-related complications [4]. In comparison to first-generation DP-DES, early-generation BP-DES demonstrated superior safety and a decrease in patient-centered outcomes. However, it has been demonstrated that more recent generations of durable polymers are thromboresistant and even safer than BMS [5]. PF-DES was developed to provide similar benefits to BMS (lower chances of stent thrombosis) and DP-DES (less risk of lesion revascularization). The key obstacle for PF-DES has achieved a high enough level of the antiproliferative agent in the inorganic coating to confirm neointimal hyperplasia and in-stent restenosis inhibition [6].

There are currently conflicting prognostic data reported for PF-DES, and few randomized controlled trials (RCTs) have been conducted to compare the clinical outcomes of the polymer-free drug-eluting stent (PF-DES) approach to that of polymer-coated DES (PC-DES). In this meta-analysis, we report findings from a review of the relevant literature. Therefore, the purpose of the present study was to conduct a comprehensive meta-analysis of available randomized controlled trials (RCTs) and cohort studies, equating the effect of PF-DES versus PC-DES on patient outcomes.

## Review

Methods

Methodology

This study followed the preferred reporting items for systematic review and meta-analysis (PRISMA) [7] guidelines to ensure the highest quality results from this meta-analysis.

Search Strategy and Selection

A systematic literature search was performed on PubMed, Embase, and MEDLINE databases up until January 23, 2023, using the following subject keywords and their MeSH terms: (Polymer-free drug-eluting stents OR PF-DES) AND (Polymer-coated drug-eluting stents OR PC-DES) AND (Coronary artery disease OR Ischemic heart disease OR CAD). Appendix 1 summarizes the detailed search approach. MK and SK independently evaluated the search results. In the event of disagreement, a third reviewer (KM) was consulted. The eligibility of studies was initially determined based on the study's title and abstract; then, the full text was evaluated. In addition, the references of the chosen studies were meticulously examined.

Study Inclusion and Exclusion Criteria

Inclusion criteria: The inclusion criteria for studies in this research involved comparative analyses between PF-DES and PC-DES that use either permanent or bioresorbable polymer coatings. Additionally, studies must have complete clinical and outcome data available to ensure accurate assessments of the effectiveness of each type of drug-eluting stent. Only studies that meet these inclusion criteria were considered for this research.

Several exclusion criteria had been established to ensure the integrity and reliability of the study results: First, follow-up data must be available for at least 90% of patients. Second, ongoing studies or studies with irretrievable data will be excluded. Third, bare metal stents in the control group will not be accepted. Finally, studies without clinical outcome endpoints will also be excluded. By applying these exclusion criteria, the study will only consider high-quality research with complete and reliable data that can provide valid conclusions regarding comparative studies between PF-DES and polymer-coated DES (PC-DES) with either permanent or bioresorbable polymer. This approach will help ensure that the results obtained from this study are accurate and trustworthy and can be used to guide future clinical decision-making. Reviews, editorials, protocols, case reports, and studies lacking a comparison and outcome were excluded. No language restrictions were enforced.

Data Extraction

Two researchers independently extracted the data (SK and MK). If data needed to be completed or clarified, the authors were contacted. Disputes were resolved through consensus. The data were managed following the principle of intention-to-treat.

Data extraction from the relevant studies included: the first author, year of publication, study type (cohort or randomized controlled trial), study follow-up duration, the total number of patients with coronary artery disease (CAD), and the number of patients in each group (PF-DES and PC-DES). Also extracted were baseline characteristics such as age, gender, body mass index (BMI), history of myocardial infarction (MI), history of stroke, and the number of vessels involved. The primary outcomes of all-cause, cardiovascular, and non-cardiovascular mortality were extracted from the tables and text of the individual studies after a thorough examination. MI, stent thrombosis, target lesion revascularization (TLR), target vessel revascularization (TVR), target lesion failure, target vessel failure, stroke, and major adverse cardiovascular events were secondary outcomes (MACE).

Assessment of Risk of Bias

All observational studies were evaluated using the Newcastle-Ottawa scale [8], while randomized controlled trials were assessed using the Cochrane risk of bias tool [9].

Data Analysis

Only comparative studies were analyzed statistically using Review Manager 5.4.1 (The Nordic Cochrane Center, The Cochrane Collaboration, 2014, Denmark) and comprehensive meta-analysis. This meta-analysis provides a pooled effect of relative risks (RRs) for dichotomous outcomes and weighted mean differences (WMDs) for continuous outcomes calculated utilizing the generic-inverse variance with a random-effects model. Forest plots were used to display the results of pooled analyses. To assess publication bias, funnel plots were constructed for each primary outcome. Low (25%), moderate (25-75%), and high (>75%) levels of heterogeneity were determined using the Higgins I^2^ test [10]. The association between baseline variables such as age, male gender, and previous myocardial infarction and outcomes such as death from all causes and cardiovascular death was investigated using univariate meta-regression. All analyses were considered significant if the p-value was less than 0.05. Since the data were compiled and synthesized from earlier clinical trials for which the researchers had already received informed consent, no ethics committee approval was required for this study.

Results

Eligible Studies

**Figure 1:**
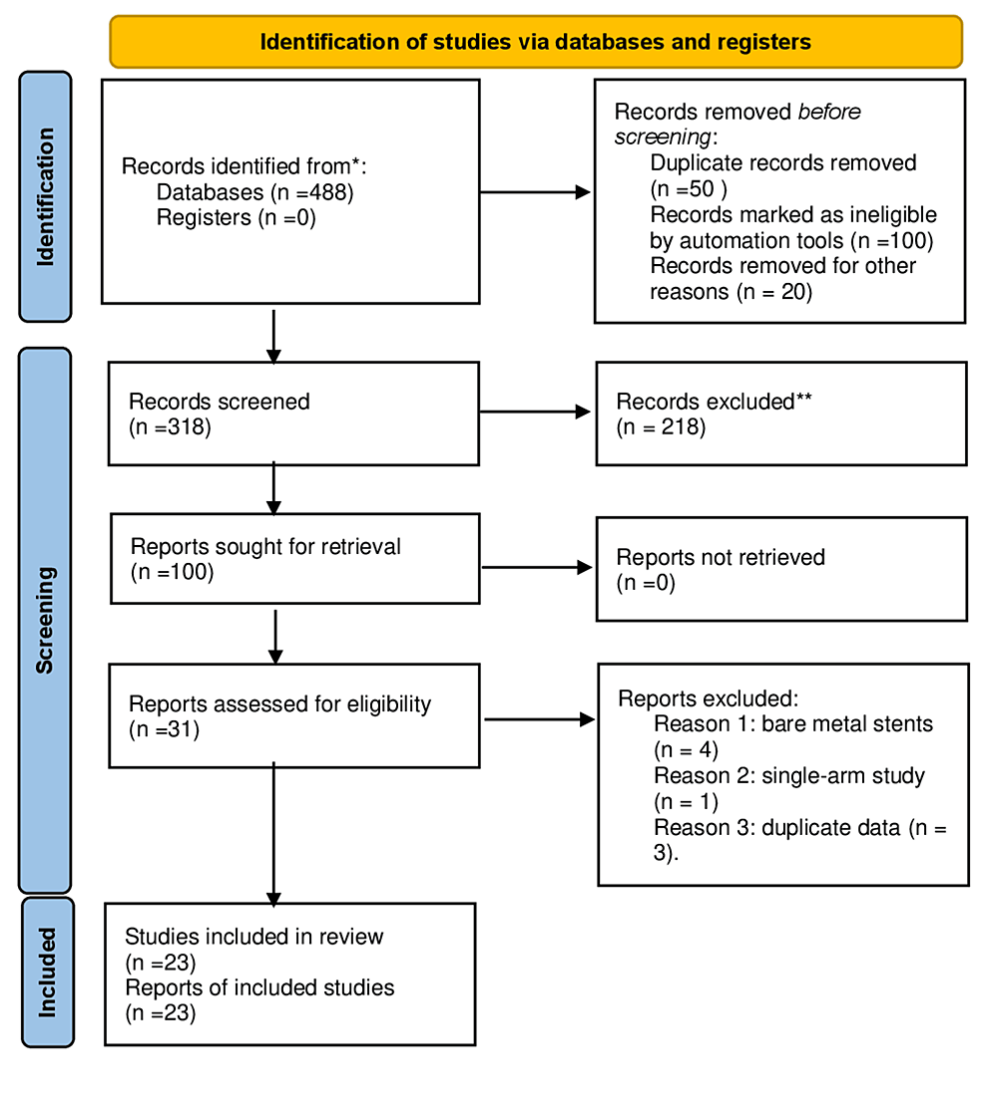
PRISMA flow chart. PRISMA: preferred reporting items for systematic review and meta-analysis.

As depicted in the PRISMA flowchart in Figure 1, a total of 488 studies were screened for inclusion in the meta-analysis. Four studies that compared PF-DES to bare metal stents were omitted (BMS) [11-14]. One study was excluded as it was a single-arm study with no control group [15]. After excluding studies with duplicate data [16-18] and conducting a meta-analysis, we finally included 23 studies [19-41], which comprised of 20 RCTs and three observational studies with a total of 28,555 patients. There were 14,951 (52.3%) patients assigned to a PF-DES strategy and 13,566 (47.5%) patients assigned to a PC-DES approach. In most studies, PF-DES was compared to permanent-polymer (PP) DES. Three studies compared PF-DES to bioresorbable polymer DES (BP-DES), whereas two studies randomized participants to either BP or PP-DES. Seven trials permitted the inclusion of ST-elevation myocardial infarction (STEMI) patients, with one study comprising the entire population, whereas 16 studies excluded patients with acute MI. Two clinical trials were conducted on people at increased risk, including all diabetic patients. Only preliminary data were available for one study, whereas full-text manuscripts were available for the remaining 15 studies. In all studies, patients received a dual antiplatelet therapy (DAPT) regimen lasting at least six months. Appendix 2 displays the study characteristics of the included trials. The duration of follow-up varied widely, from 12 months to 60 months (median 24 months).

Baseline Characteristics of Patients

The characteristics of the included patients at the outset are listed in Table 1. Most of the patients included in the study were middle-aged or older men who smoked and who suffered from conditions like diabetes, hypertension, and a previous myocardial infarction.

**Table 1:** Baseline characteristics. PF-DES: polymer-free drug-eluting stent, PC-DES: polymer-coated drug-eluting stent, N/A: not available, SD: standard deviation, MI: myocardial infarction, PCI: percutaneous coronary intervention, RCT: randomized control trial.

Study (year)	Study design	Total no. of patients	No. of patients		Age (mean ± SD)		Male no. (%)		Diabetes no. (%)		Hypertension no. (%)		Hyperlipidemia no. (%)		Current smoker no. (%)		Previous MI no.(%)		Previous PCI no. (%)	
			PF-DES	PC-DES	PF-DES	PC-DES	PF-DES	PC-DES	PF-DES	PC- DES	PF-DES	PC- DES	PF-DES	PC- DES	PF-DES	PC- DES	PF- DES	PC- DES	PF-DES	PC- DES
Costa et al. (2016) [19]	Multicenter RCT	182	122	60	66.8 ± 9.2	67.9 ± 8.0	87 (71.3)	40 (66.7)	35 (28.6)	15 (25)	104 (85.2)	51 (85)	86 (70.4)	45 (75)	22 (18)	7 (12.3)	25 (20.4)	11 (18.3)	46 (37.7)	27 (45.8)
Carie et al. (2012) [20]	Multicenter RCT	323	162	161	64.9 ± 10.2	64.3 ± 10.4	124 (76.5)	109 (67.7)	48 (29.6)	39 (24.2)	104 (64.2)	104 (64.6)	102 (63)	98 (60.9)	39 (24.1)	40 (24.8)	14 (8.6)	15 (9.3)	26 (16.0)	23 (14.3)
Dang et al. (2012) [21]	RCT	105	50	55	65.2 ± 13.8	67.1 ± 12.5	34 (68)	39 (70.9)	12 (24)	15 (27.3)	23 (46)	23 (41.8)	10 (20)	13 (23.6)	33 (66)	34 (61.8)	2 (4)	4 (7.3)	1 (2)	1 (1.8)
Chen et al. (2013) [22]	Multicenter RCT	346	173	173	63.4 ± 10.4	64.2 ± 11.3	136 (78.6)	135 (78.0)	50 (28.9)	46 (26.7)	121 (69.9)	116 (67.1)	51 (29.5)	55 (31.8)	50 (28.9)	46 (26.7)	20 (11.6)	32 (18.5)	28 (16.2)	39 (22.5)
King et al. (2013) [23]	Multicenter RCT	450	225	225	66.8 ± 10.5	66.6 ± 10.2	169 (75.1)	177 (78.6)	73 (32)	58 (26)	142 (63)	155 (69)	165 (73)	170 (76)	43 (19)	39 (17)	72 (32)	71 (32)	N/A	N/A
Byrne et al. (2010) [24]	Multicenter RCT	1007	333	674	67.0 ± 11.2	66.9 ± 11	257 (77.1)	515 (76.4)	96 (28.8)	180 (26.7)	229 (64.9)	443 (65.7)	209 (62.8)	653 (67.2)	66 (19.8)	119 (17.6)	84 (25.2)	188 (27.8)	N/A	N/A
Byrne et al. (2009) [25]	Multicenter RCT	605	201	404	66.8 ± 9.70	65.7 ± 11.1	157 (78.1)	323 (79.9)	55 (27.2)	111 (27.4)	135 (67.2)	275 (68)	143 (71.1)	273 (67.5)	36 (17.8)	63 (15.6)	66 (32.9)	133 (32.9)	N/A	N/A
Massberg et al. (2011) [26]	Multicenter RCT	3002	2002	1000	67.7 ± 11.2	68.1 ± 10.8	1532 (76.5)	763 (76.3)	575 (28.7)	295 (29.5)	1336 (66.7)	666 (66.6)	1257 (62.8)	650 (65.0)	357 (17.8)	166 (16.6)	586 (29.3)	299 (29.9)	N/A	N/A
Stiermaier et al. (2011) [27]	Multicenter RCT	236	120	136	67.0 ± 9.5	67.3 ± 9.1	83 (69)	79 (68)	120 (100)	116 (85)	118 (98)	112 (97)	N/A	N/A	28 (23)	31 (27)	26 (22)	26 (22)	38 (32)	33 (28)
Rozemeijer et al. (2018) [28]	Multicenter RCT	1491	747	744	64∙7 ± 11∙3	65∙1 ± 10∙6	565 (75∙6)	577 (77∙6)	155 (20∙8)	149 (20∙0)	412 (55∙2)	411 (55∙2)	325 (43∙5)	340 (45∙8)	193 (25∙9)	191 (25∙7)	139 (18∙6)	158 (21∙2)	138 (18∙5)	166 (22∙3)
Romaguera et al. (2016) [29]	Multicenter RCT	112	56	56	66.7 ± 9.8	67.2 ± 8.8	45 (0.80)	39 (0.69)	56 (100)	56 (100)	46 (82.1)	49 (87.5)	45 (80.4)	47 (83.9)	30 (53.6)	35 (62.5)	13 (23.2)	17 (30.4)	22 (39.3)	19 (33.9)
Shiratori et al. (2014) [30]	Multicenter RCT	164	84	80	65.9 ± 8.0	67.2±10.5	64 (76.2)	55 (68.8)	31 (36.9)	22 (27.5)	61 (72.6)	59 (73.8)	51 (60.7)	50 (62.5)	17 (20.2)	14 (17.5)	24 (28.6)	27 (33.8)	23 (27.4)	24 (30.4)
Okkels et al (2018) [31]	Multicenter RCT	3151	1572	1579	66.4 ± 10.7	66.1 ± 11.1	1219 (77.5)	1219 (77.5)	303 (19.2)	304 (19.3)	850 (56.0)	893 (59.0)	830 (55)	830 (55)	443 (29.8)	437 (29.3)	224 (14.7)	234 (15.2)	322 (20.9)	311 (20.9)
Zhang et al. (2013) [32]	Single center RCT	648	327	321	65.2 ± 10.4	65.8 ± 11.1	214 (65.4)	220 (68.5)	83 (25.3)	89 (27.7)	211 (64.5)	209 (65.1)	114 (34.8)	114 (35.51)	134 (40.9)	125 (38.9)	16 (4.89)	15 (4.67)	26 (7.95)	38 (11.8)
Natsuaki et al. (2013) [33]	Multicenter RCT	3235	1617	1618	69.1 ± 9.8	69.3 ± 9.8	1245 (77)	1253 (77)	745 (46)	740 (46)	1317 (81)	1323 (82)	1265 (78)	1263 (78)	301 (19)	293 (18)	460 (28)	454 (28)	816 (50)	820 (51)
Zhang et al. (2014) [34]	Multicenter RCT	291	143	148	55.3 ± 10.7	59.5 ± 9.8	106 (0.74)	117 (0.79)	22 (15.3)	27 (18.2)	81 (56.6)	75 (50.6)	38 (26.5)	52 (35.1)	77 (53.8)	73 (49.3)	53 (37)	35 (23.6)	18 (12.5)	22 (14.8)
Windecker et al. (2020) [35]	Multicenter RCT	1996	993	1003	74.1 ± 9.8	74.0 ± 9.5	563 (65.7)	677 (67.4)	382 (38.5)	388 (38.7)	807 (81.3)	796 (79.4)	619 (62.3)	643 (64.1)	108 (10.9)	93 (9.4)	249 (25.1)	264 (26.3)	230 (23.2)	237 (23.6)
Gregersen et al. (2022) [36]	Multicenter RCT	3151	1572	1579	66.4 ± 10.7	66.1 ± 11.1	1219 (77.5)	1221 (77.3)	304 (19.3)	303 (19.2)	893 (59.0)	850 (56.0)	830 (55)	777 (51.5)	443 (29.8)	437 (29.3)	224 (14.7)	234 (15.2)	322 (20.9)	311 (20.9)
Hemert et al. (2021) [37]	Multicenter RCT	1491	721	712	64.7 ± 11.3	65.1 ± 10.6	565 (75.6)	577 (77.6)	155 (20.8)	149 (20.0)	412 (55.2)	411 (55.2)	325 (43.5)	340 (45.8)	193 (25.9)	191 (25.7)	139 (18.6)	158 (21.2)	138 (18.5)	166 (22.3)
Rozemeijer et al. (2019) [38]	Prospective registry	734	361	373	66.5 ± 11.8	66.8 ± 12.7	272 (72.9)	233 (64.5)	112 (30.0)	93 (25.8)	221 (59.6)	205 (57.1)	156 (42)	145 (40.3)	128 (34.3)	143 (39.6)	95 (25.5)	91 (27.3)	117 (31.5)	98 (27.3)
Gallone et al. (2021) [39]	Multicenter observational	1169	440	729	71 ± 11	68 ± 11	326 (74.1)	556 (76.3)	125 (28.4)	214 (29.4)	338 (76.8)	543 (74.5)	262 (59.5)	399 (54.7)	175 (39.8)	339 (46.5)	93 (21.2)	226 (31.0)	108 (24.7)	219 (30.0)
Loewenstein et al. (2022) [41]	Prospective registry	1664	928	736	N/A	N/A	771 (83)	612 (83)	303 (38.7)	235 (37.6)	510 (64.3)	416 (65.9)	621 (78.9)	513 (81.2)	263 (33.9)	244 (39.5)	201 (26)	166 (26.9)	352 (37.9)	292 (39.7)
Koch et al. (2021) [40]	Multicenter RCT	3002	2002	1000	67.8 ± 10.2	68.4 ± 10.9	1532 (76.5)	763 (76.3)	870 (43.4)	213 (21.3)	1336 (66.7)	666 (66.6)	1257 (62.7)	650 (65)	357 (17.8)	166 (16.6)	586 (29.2)	299 (29.9)	N/A	N/A

Quality Assessment and Publication Bias

**Figure 2:**
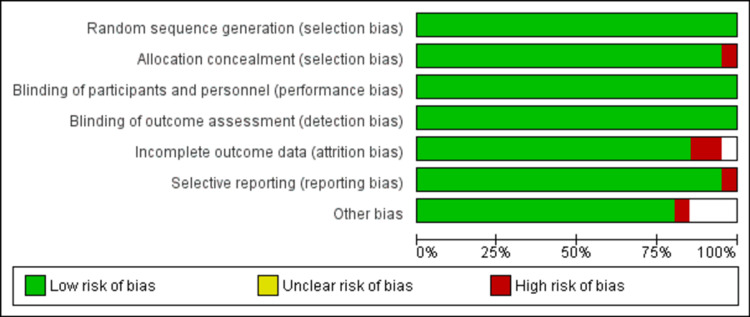
Cochrane risk of bias tool for assessing publication bias in randomized controlled trials.

**Figure 3:**
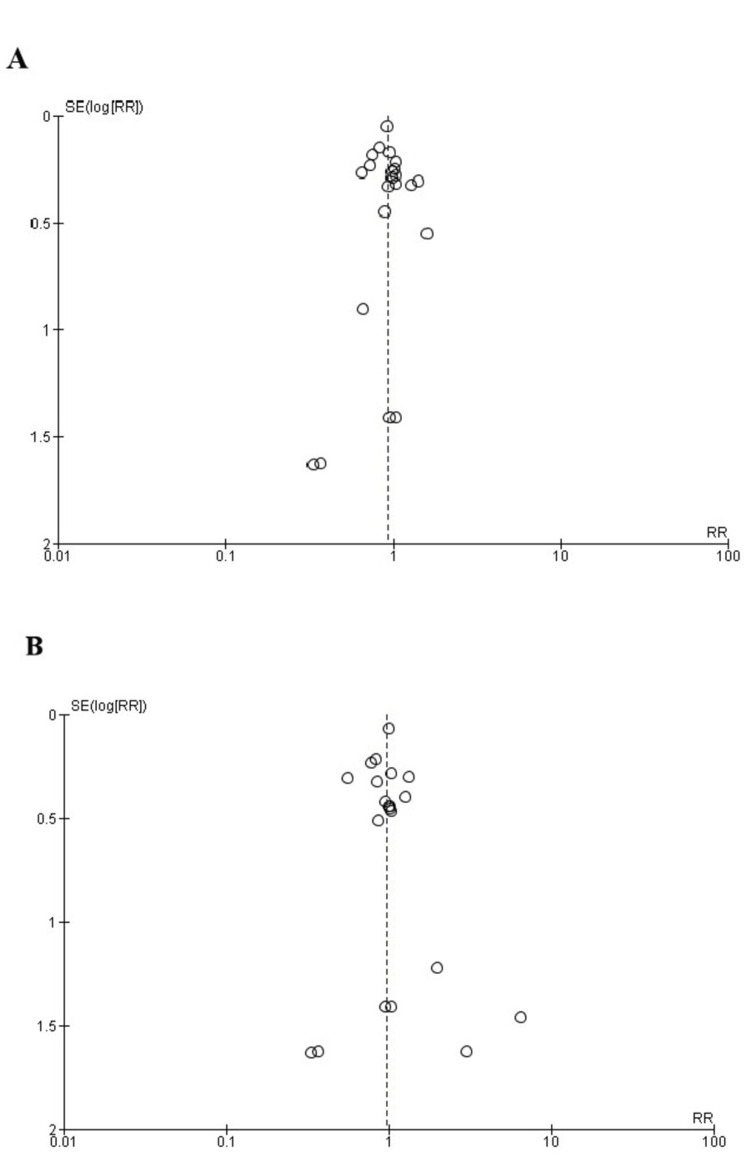
Funnel plot of (a) death from all causes, (b) cardiovascular death. SE: standard error, RR: relative risk.

**Figure 4:**
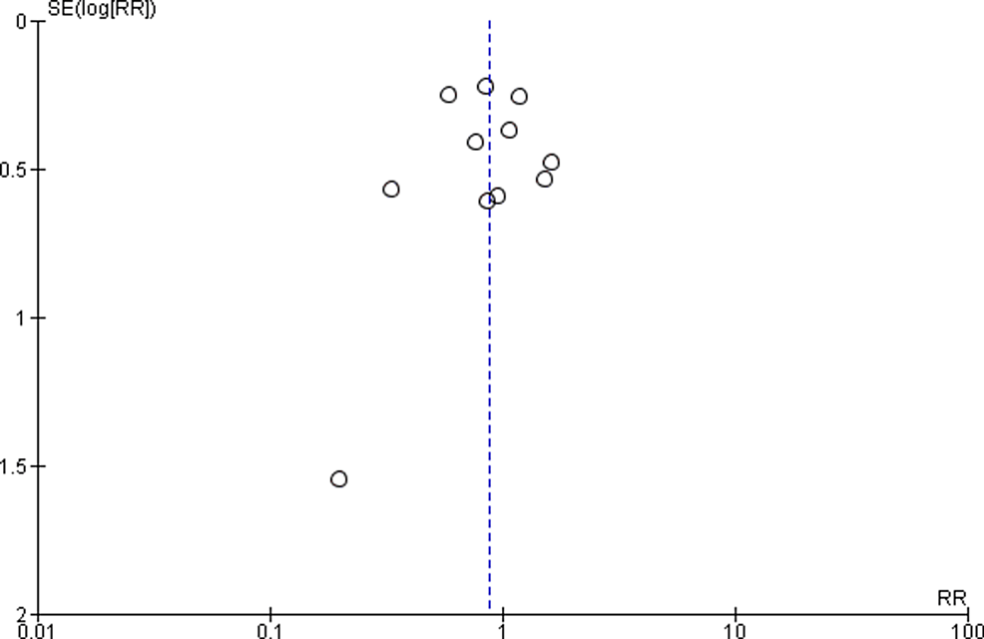
Funnel plot of non-cardiovascular death. SE: standard error; RR: relative risk.

The Newcastle-Ottawa scale, a tool used to assess study quality, discovered a low likelihood of bias in observational studies, as shown in Table 2. Using the Cochrane method of assessing RCTs, we found trials of medium-to-high quality, as shown in Figure 2. The results were unaffected by publication bias, as demonstrated by the funnel plots as shown in Figures 3, 4.

**Table 2:** Newcastle-Ottawa scale to assess publication bias in observational studies. The Newcastle-Ottawa scale quality instrument is scored by awarding a point for each answer that is marked with an asterisk below. Possible total points are four points for selection, two points for comparability, and three points for outcomes. Good quality: three or four stars in the selection domain and one or two stars in the comparability domain and two or three stars in the outcome/exposure domain fair quality: two stars in the selection domain and one or two stars in the comparability domain and two or three stars in outcome/exposure domain poor quality: zero or one star in selection domain or zero stars in comparability domain or zero or one stars in outcome/exposure domain.

Study	Selection				Comparability	Outcomes			Total
	Representativeness of the exposed cohort	Selection of the non-exposed cohort	Ascertainment of exposure	Demonstration that outcome of interest was not present at the start of the study	Comparability of cohorts on the basis of the design or analysis	Assessment of outcome	Was follow-up long enough for outcomes to occur	Adequacy of follow-up of cohorts	
Rozemeijer et al. (2019) [38]	*	*	*	*	**	*	*	*	*********
Gallone et al. (2021) [39]	*	*	*	*	*	*	*	*	********
Loewenstein et al. (2022) [41]	*	*	*	*	**	*	*	*	*********

Primary Outcomes

**Figure 5:**
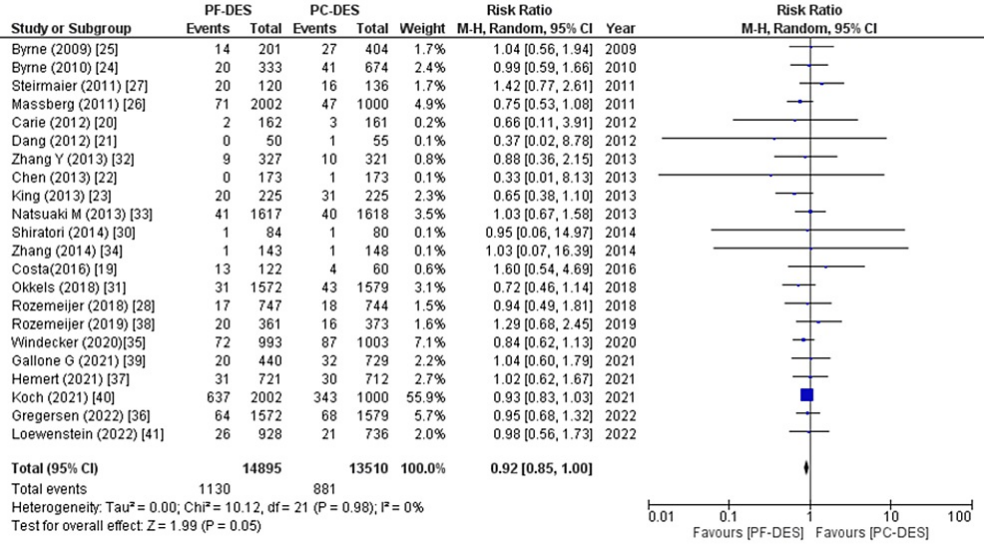
Forest plot of death from all causes. RR: relative risk, CI: confidence interval, PF-DES: polymer-free drug-eluting stent, PC-DES: polymer-coated drug-eluting stent. Sources: References [19-28,30-41].

**Figure 6:**
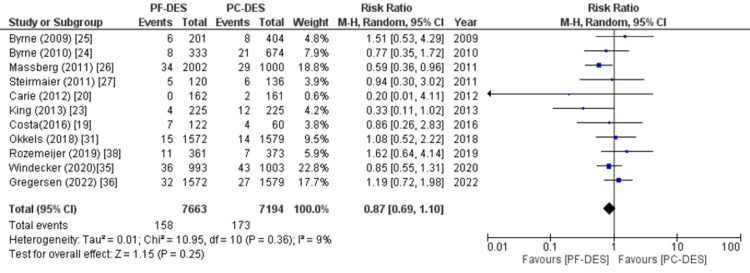
Forest plot of non-cardiovascular death. RR: relative risk, CI; confidence interval, PF-DES: polymer-free drug-eluting stent, PC-DES: polymer-coated drug-eluting stent. Sources: References [19,20,23-27,31,35,36,38].

**Figure 7:**
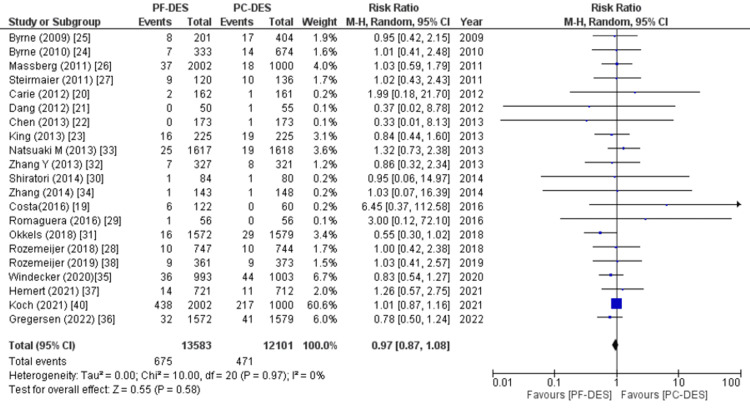
Forest plot of cardiovascular death. RR: relative risk, CI: confidence interval, PF-DES: polymer-free drug-eluting stent, PC-DES: polymer-coated drug-eluting stent. Sources: References [19-38,40].

All-cause mortality, cardiovascular mortality, and non-cardiovascular mortality were the primary endpoints. Twenty-two out of the twenty-three studies examined deaths from all causes, and the pooled analysis found a marginally lower risk of death with PF-DES compared to PC-DES (RR (95% CI) = 0.92 (0.85, 1.00), p = 0.05, I^2^ = 0%), as shown in Figure 5. Relative risk (RR) between PF-DES and PC-DES for non-cardiovascular mortality was also observed in a meta-analysis (RR (95% CI) = 0.87 (0.69, 1.10), p = 0.25, I^2^ = 9%) as shown in Figure 6, with data from 11 studies. There was also no significant difference between the groups when it came to cardiovascular mortality (RR (95% CI) = 0.97 (0.87, 1.08), p = 0.58, I^2^ = 0%) as shown in Figure 7, which was reported in 21 studies.

Secondary Outcomes

**Figure 8:**
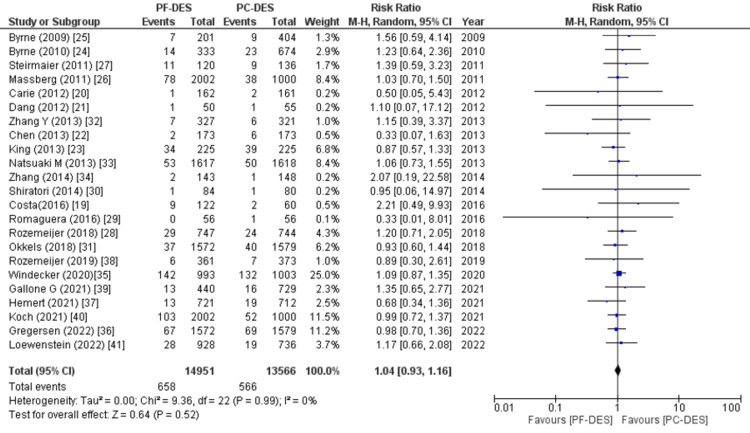
Forest plot showing the rate of myocardial infarction (MI). RR: relative risk, CI: confidence interval, PF-DES: polymer-free drug-eluting stent, PC-DES: polymer-coated drug-eluting stent. Sources: References [19-41].

Myocardial infarction: All 23 studies reported the number of patients who experienced myocardial infarction following treatment in the follow-up period, and pooled analysis revealed that there was no significant difference between the two groups (RR (95% CI) = 1.04 (0.93, 1.16), p = 0.52, I^2^ = 0%) as shown in Figure 8.

**Figure 9:**
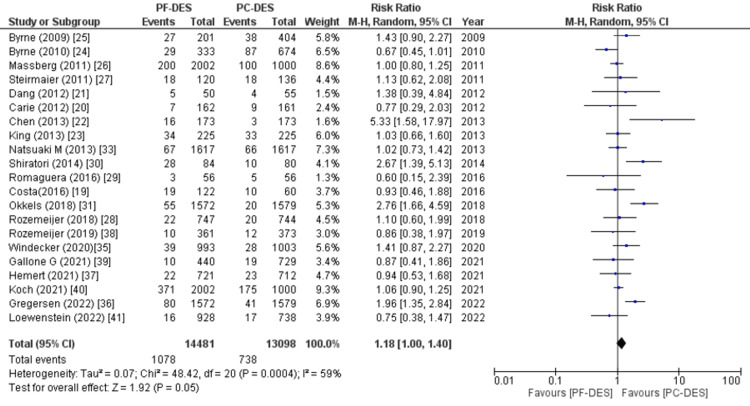
Forest plot showing the rate of stent thrombosis. RR: relative risk, CI: confidence interval, PF-DES: polymer-free drug-eluting stent, PC-DES: polymer-coated drug-eluting stent. Sources: References [19-41].

Stent thrombosis: All 23 studies reported the number of patients who developed stent thrombosis in the follow-up period, and the pooled analysis revealed a marginally increased risk of stent thrombosis for PF-DES compared to PC-DES (RR (95% CI) = 1.12 (0.93, 1.35), p = 0.23, I^2^ = 0%) as shown in Figure 9.

**Figure 10:**
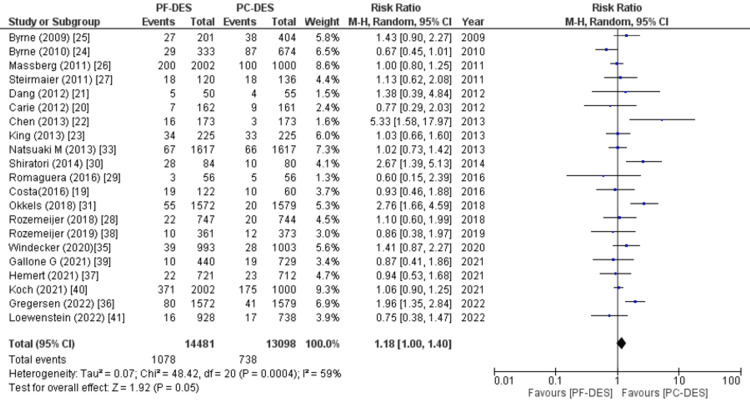
Forest plot showing target lesion revascularization (TLR). RR: relative risk, CI: confidence interval, PF-DES: polymer-free drug-eluting stent, PC-DES: polymer-coated drug-eluting stent. Sources: References [19-31,33,35-41].

**Figure 11:**
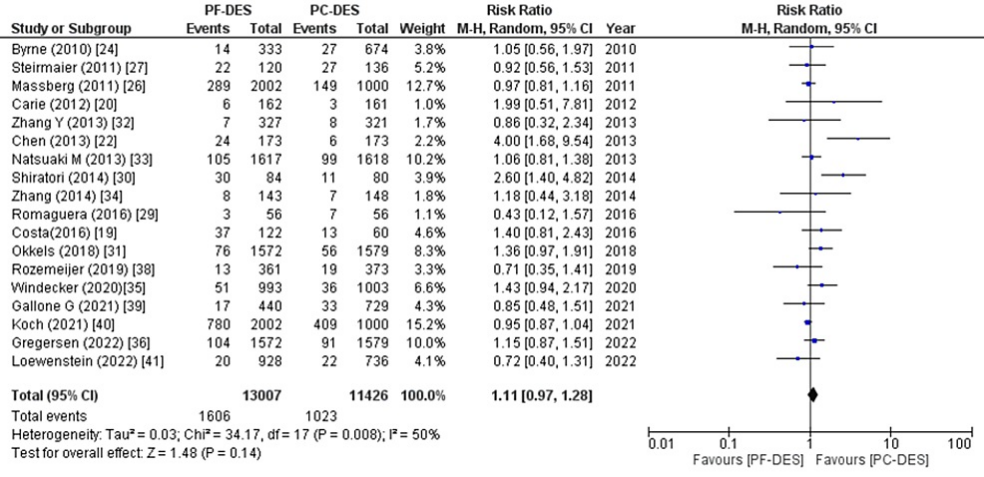
Forest plot showing target vessel revascularization (TVR). RR: relative risk, CI: confidence interval, PF-DES: polymer-free drug-eluting stent, PC-DES: polymer-coated drug-eluting stent. Sources: References [19,20,22,24,26,27,29-36,38-41].

Target lesion and target vessel revascularization: Data on target lesion and target vessel revascularization were reported by 21 and 18 studies, respectively, and the pooled analysis demonstrated that PF-DES was associated with slightly higher rates of revascularization than PC-DES (RR (95% CI) = 1.18 (1.00, 1.40), p = 0.05, I^2^ = 59%) (RR (95% CI) = 1.11 (0.97, 1.28), p = 0.14, I^2^ = 50%) as shown in Figures 10, 11.

**Figure 12:**
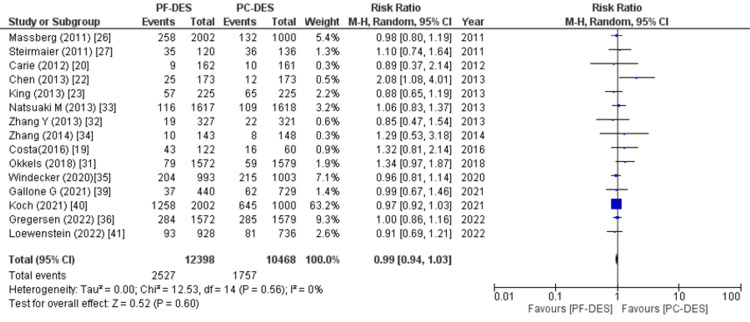
Forest plot showing major adverse cardiovascular events (MACEs). RR: relative risk, CI: confidence interval, PF-DES: polymer-free drug-eluting stent, PC-DES: polymer-coated drug-eluting stent. Sources: References [19,20,22,23,26,27,31-36,39-41].

Major adverse cardiovascular events: 15 of 23 studies reported data on major cardiovascular adverse events, and pooled analysis revealed no significant difference between the two groups (RR (95% CI) = 0.99 (0.94, 1.03), p = 0.60, I^2^ = 0%) as shown in Figure 12.

**Figure 13:**
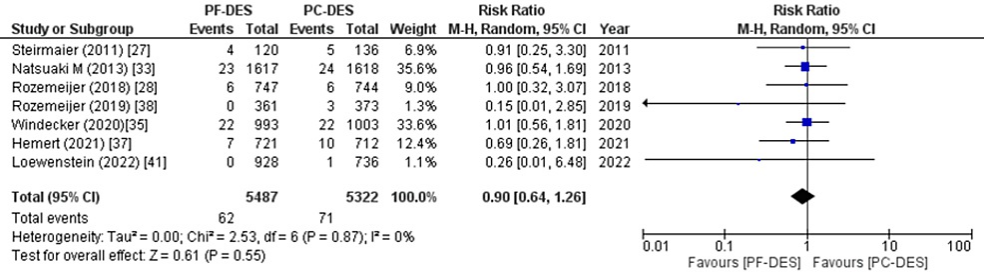
Forest plot showing the rate of stroke. RR: relative risk, CI: confidence interval, PF-DES: polymer-free drug-eluting stent, PC-DES: polymer-coated drug-eluting stent. Sources: References [27,28,33,35,37,38,41].

Stroke: Seven of 23 studies reported data on the number of patients who suffered a stroke during the follow-up period, and the pooled analysis revealed a statistically insignificant risk of stroke during the follow-up period with PF-DES versus PC-DES (RR (95% CI) = 0.90 (0.64, 1.26), p = 0.55, I^2^ = 0%) as shown in Figure 13.

Effect of age, male gender, and previous myocardial infraction on death from all causes and cardiovascular death

According to a univariate meta-regression shown in Table 3, male gender and a history of myocardial infarction (MI) are independently associated with an increased risk of death from all causes and cardiovascular death, while age has no effect.

**Table 3:** Univariate meta-regression showing the effect of age, male gender, and history of MI on death from all causes and cardiovascular death. MI: myocardial infarction.

Outcomes	Covariates	Co-efficient	p-value
Death from all causes	Age	−0.0023	0.4524
	Male gender	8.23	0.975
	Previous MI	7.81	0.9813
Cardiovascular death	Age	0.0085	0.3659
	Male gender	10.14	0.96
	Previous MI	9.06	0.95

Discussion

Almost 28,555 participants were included in 20 randomized controlled trials and three observational studies for this recent meta-analysis evaluating PF-DES versus PC-DES. We found that PF-DES lowered mortality relative to conventional DES and that this improvement was independent of preventing large recurrent ischemia episodes, but on the other hand, it also increased the risk of stent thrombosis.

The emergence of drug-eluting stents (DES) transformed the care of coronary heart disease, with substantial declines in adverse outcomes following percutaneous coronary intervention (PCI), particularly in comparison to balloon angioplasty and bare metal stents (BMS). There is concern that polymer coatings, which are necessary for efficient drug release, may cause localized inflammation within the coronary segment to be treated, increasing the likelihood of prolonged healing time and thrombotic complications. Drug-eluting stents (DES) have an evident advantage over conventional bare metal stents in reducing the rate of recurring revascularization, but this advantage has been offset by a higher rate of late thrombotic events and restenosis [42]. Nonpolymeric drug-coated stents have been developed as a replacement for biodegradable and long-lasting polymeric DES. However, the absence of a drug carrier has been associated with decreased effectiveness in inhibiting neointimal hyperplasia, most likely due to inadequate or uncontrolled drug delivery at the target coronary site [18].

Polymer-free biofilms A9-coated BioFreedom stent and the ultrathin strut biodegradable polymer sirolimus-eluting Orsiro stent were compared for the first time in the SORT OUT IX trial [18]. There was no statistically significant difference between the two stents on the composite target lesion failure (TLF) endpoint at the two-year follow-up. TLR risk persisted for an additional two years in the BioFreedom stent group. The risk of TLR was similar between groups in the second year after implant placement, while the risk of medically driven TLR was larger during the first year [36]. Different DES technologies used by the BioFreedom stent and the other stents tested resulted in a higher TLR rate for the former. Drug release from the study stent occurs at a different rate than from other stents. BioFreedom stents, which are polymer-free, release 90% of their drug within 48 hours. The Orsiro stent is coated in a polymer made of silicon carbide, which breaks down over the course of 12 to 24 months to release the drug gradually over the course of three months. Second, the BioFreedom stent's struts are thicker (120 m) than the Orsiro's (60-80 m), and this is known to affect restenosis risk [35,36].

Numerous studies have examined the causes of these post-DES implant delayed events and found that stent features and fundamental health factors, such as older age, diabetes, and acute manifestations, may operate as independent risk factors of late stent consequences. Ullah et al. [43] suggested that patients who received either PF-DES or PC-DES appeared to have a similar risk of major adverse cardiovascular events (MACEs), strokes, MI, stent thrombosis, and the need for target lesion and vessel revascularization (TLR and TVR) [43]. Their overall results were consistent across multiple subgroup analyses based on the length of follow-up (one month to 10 years), the presence of diabetes mellitus (DM), the clinical presentation (angina vs. STEMI), and the kind of drug-eluting PF stents used. In comparison to PC-DES, the odds of overall non-cardiovascular and all-cause mortality in PF-DES were 22% and 13% lower, respectively. Regardless of the clinical presentation (angina or acute coronary syndrome (ACS)) or history of diabetes mellitus (DM), these observations were most emphasized at long follow-up intervals and in patients receiving rapamycin plus probucol eluting PF stents. In all of the foregoing predetermined subgroup analyses, there was no longer a difference between the two groups in mortality or any other clinical outcomes.

In an investigation, 3002 people with coronary heart disease were given either polymer-free sirolimus- and probucol-eluting stents (PF-SES: n = 2002) or durable zotarolimus-eluting stents (DP-ZES: n = 1000) as their treatment. In the randomized Intracoronary Stenting and Angiographic Restenosis-Test Equivalence Between Two Drug-Eluting Stents (ISAR-TEST) 5 trial, the rates of all-cause mortality, any myocardial infarction, and any revascularization were significant but similar in patients with diabetes mellitus handled with PF-SES as compared to DP-ZES (74.8% vs. 79.6%; P = 0.08; hazard ratio 0.86; 95% CI 0.73-1.02) and patients without diabetes mellitus (PF-SES 62.5% vs. DP-ZES 62.2%; P = 0.88; hazard ratio 0.99; 95% CI 0.88-1.11) [44]. Death rates were lower in the 3151 patients who participated in the SORT OUT IX trial [18], which equated PF-DES with the coming generation of BP-sirolimus eluting stents, but the rate of TLR was more than doubled (3.5% vs. 1.3%), leading to a null prognostic effect. Even though PF-DES has been shown to speed up re-endothelization and decrease late-lumen loss upon angiographic re-evaluation of the stent, its effects on the outcome are still up for debate.

This recent paper of more extensive randomized clinical trials allows us to conduct the current meta-analysis, which compares PF-DES to PC-DES, including PP and BP-DES. We discovered that patients who received the new stents had a reduced all-cause mortality rate, similar to what Nogic et al. [45] discovered. However, this increase in survival was not due to a reduction in recurring ischemic episodes or cardiovascular deaths. Admittedly, our study's findings were unaffected by patients' risk profiles, as validated by meta-regression analysis or the expulsion of trials undertaken in specific patient subgroups, such as STEMI or diabetic patients. Indeed, it could be argued that the low frequency of events influenced the results of previous and current meta-analyses. In particular, 1% of patients developed stent thrombosis, which could be attributed to the inclusion of a low-risk community of stable patients with confined outgrowths of cardiovascular problems. The vast majority of trials were underwhelming in assessing actual result metrics, and the greatest benefits of PF-DES were confirmed in trials encompassing subgroups of patients at greater risk [44,45].

In a cohort of patients receiving percutaneous coronary intervention for coronary bifurcation disease, Gallone et al. [39] reported no significant difference in the risk of major adverse cardiac events (MACE) between the polymer-free-biolimus eluting stent (PF-BES) and the ZES stents at 400 days. Patients who were presented with stable coronary artery disease or non-left main (LM) bifurcation lesions had a trend towards higher rates of adverse events with the PF-BES, but these rates were not statistically significant [39]. In aspects of major adverse cardiovascular events, all-cause mortality, stent thrombosis, and target lesion revascularizations, the current analysis suggests that both PF-BES and ZES have an improved therapeutic profile when used for the cure of bifurcation lesions. Relevantly, despite being largely non-statistically significant, numerical differences were discovered in the hazard ratios of the majority of the investigated outcomes. These differences were consistent in both the primary and sub-group analyses, suggesting that they may have clinical significance [36].

We used univariate meta-regression to evaluate heterogeneity and investigate possible differences between the datasets. This study found that the male gender and a history of myocardial infarction (MI) independently contributed to a higher risk of death from all causes and cardiovascular death. At the same time, age played no role in the association. Using meta-regression analysis, Verdoia et al. [46] defined multiple correlations; nevertheless, the advantages of the more recent PFDES method were not dependent on the patient's identified risks (p = 0.91), the prevalence of diabetes mellitus (r = 0.008, p = 0.08), or the incidence of acute coronary syndromes (p = 0.14).

Study Limitations

Our study has some limitations. The first one relates to the combination of multiple trials. Although the outcomes showed no notable heterogeneity, there were differences in the baseline risk profiles of the participants included in the study, such as patients with stable and acute coronary syndromes, as well as various stent comparators in the comparison group, including older and newer generation PP-DES and BP-DES. Additionally, the analysis included fewer, more extensive trials, which may have resulted in insignificant secondary outcomes due to their low occurrence rate. Thirdly, the follow-up duration varied widely among studies, with some indicating much longer durations than others. It is preferable to conduct long-term studies when evaluating the efficacy and safety of surgical implants, particularly in heart surgery, to identify the most significant benefits and harms.

## Conclusions

Our recent meta-analysis shows that compared to conventional DES, PF-DES is associated with lower risks of all-cause mortality but no difference in the incidence of cardiovascular mortality, cerebrovascular accidents, myocardial infarction, or major adverse cardiovascular events. Future research will undoubtedly be required to explore and corroborate our study findings, particularly in specific clinical subsets such as those at higher risk of bleeding or experiencing an acute myocardial infarction.

## Appendices

Appendix 1 

**Table 4:** Detailed search strategy. PF-DES: polymer-free drug-eluting stent, PC-DES: polymer-coated drug-eluting stents.

Database	Search strategy	Results
Pubmed	((("polymer s"[All Fields] OR "polymers"[MeSH Terms] OR "polymers"[All Fields] OR "polymer"[All Fields]) AND "free"[All Fields] AND ("drug eluting stents"[MeSH Terms] OR ("drug eluting"[All Fields] AND "stents"[All Fields]) OR "drug eluting stents"[All Fields] OR ("drug"[All Fields] AND "eluting"[All Fields] AND "stent"[All Fields]) OR "drug eluting stent"[All Fields])) OR "PF-DES"[All Fields]) AND ((("polymer s"[All Fields] OR "polymers"[MeSH Terms] OR "polymers"[All Fields] OR "polymer"[All Fields]) AND ("coated"[All Fields] OR "coating"[All Fields] OR "coating s"[All Fields] OR "coatings"[All Fields]) AND ("drug eluting stents"[MeSH Terms] OR ("drug eluting"[All Fields] AND "stents"[All Fields]) OR "drug eluting stents"[All Fields] OR ("drug"[All Fields] AND "eluting"[All Fields] AND "stent"[All Fields]) OR "drug eluting stent"[All Fields])) OR "PC-DES"[All Fields]) AND ("coronary artery disease"[MeSH Terms] OR ("coronary"[All Fields] AND "artery"[All Fields] AND "disease"[All Fields]) OR "coronary artery disease"[All Fields] OR ("crime delinq"[Journal] OR "comput aided des"[Journal] OR "cad"[All Fields]))	285
Embase	(polymer free drug eluting stent OR PF-DES) AND (polymer coated drug eluting stent OR PC-DES) AND (Coronary artery disease OR CAD)	81
Medline	(polymer-free drug eluting stent OR PF-DES) AND (polymer coated drug eluting stent OR PC-DES) AND (Coronary artery disease OR CAD)	122

Appendix 2 

**Table 5:** Study characteristics. RCT: randomized clinical trial, PF: polymer-free, PC: polymer-coated, DES: drug-eluting stents, PP: permanent polymer, BP: bioresorbable polymer, SES: sirolimus-eluting stent, PES: paclitaxel-eluting stent, BES: biolimus eluting stent, ZES: zotarolimus-eluting stent, AES: amphilimus eluting stent, UES: umerolimus eluting stent, LVEF: left ventricular ejection fraction, PCI: percutaneous coronary intervention, DEB: drug-eluting balloon; DM: diabetes mellitus; TAVI: transcatheter aortic valve implantation.

First author and year	Publication type	Stent strategy		Inclusion criteria	Exclusion criteria
		PF-DES type	PC-DES type		
Costa et al. (2016) [19]	Multi-RCT	PF-BES	PP-PES	Age >18, symptoms of stable or unstable angina, positive functional test for ischemia, single de novo target lesion, <14 mm in length, with stenosis 50% to 99%, acceptable candidate for coronary artery bypass.	Myocardial infarction, calcification, target lesion, thrombus, left ventricular ejection fraction, hypersensitivity to antithrombotic therapy, concurrent medical condition, life expectancy <18 months.
Carrie et al. (2012) [20]	Multi-RCT	PF-SES	PP-PES	Stable or unstable angina, single de novo lesions, maximum two different coronary arteries, 3.0-3.75 mm diameter.	Percutaneous coronary intervention within 30 days, acute myocardial infarction, renal failure, left ventricular ejection fraction <30%, or other comorbidities.
Dang et al. (2012) [21]	RCT	PF-PES	PP-PES	STEMI	-
Chen et al. (2013) [22]		PF-SES	BP-SES	>18 years old, signs or symptoms of myocardial ischemia, >70% diameter stenosis in the native coronary artery as evaluated by visual estimate.	RVD <2.5 mm, bifurcation lesions requiring two-stent techniques, calcification requiring directional rotablation, thrombus-containing lesions, pregnancy, renal/liver dysfunction, left ventricular ejection fraction <30%, acute myocardial infarction (MI), cardiogenic shock, blood platelet count <109 × 10^9^/L, life expectancy <12 months, allergy to any of the studied drugs, previous ischemic stroke within six months.
King et al. (2013) [23]	Multi, RCT	PF-SES	PP-PES	De novo stenosis of a native coronary artery >50% in those over 18 years old.	Target lesion of the left main stem, cardiogenic shock, myocardial infarction, malignancy, contraindications to main study medications, pregnancy.
Byrne et al. (2010) [24]	Multi-RCT	PF-sirolimus- and probucol-eluting stent	PP-ZES	Ischemic symptoms or evidence of myocardial ischemia; 50% de novo stenosis in native coronary vessel.	Patients with a life expectancy <12 months, allergy to study medications, pregnancy, or positive pregnancy test.
Byrne et al. (2009) [25]	Multi-RCT	PF-SES	PP and BP-SES	Ischemic symptoms or evidence of myocardial ischemia; 50% de novo stenosis in native coronary vessel.	Patients with a life expectancy <12 months, allergy to study medications, pregnancy, or positive pregnancy test.
Massberg et al. (2011) [26]	Multi-RCT	PF-sirolimus- and probucol-eluting stent	PP-ZES	Ischemic symptoms or evidence of myocardial ischemia; 50% de novo stenosis in native coronary vessel.	Patients with a life expectancy <12 months, allergy to study medications, pregnancy, or positive pregnancy test.
Stiermaier et al. (2011) [27]	Multi-RCT	PF-SES	PP-PES	Angina pectoris, de novo stenosis, diabetes mellitus.	Total occlusions, target lesions, in stentor bypass graft stenoses, contraindications, allergy to contrast medium, acute myocardial infarction, severe comorbidities, coagulopathy, pregnancy.
Rozemeijer et al. (2018) [28]	Multi-RCT	PF-SES	PP-ZES	>18 Years; clinical evidence of ischemic heart disease requiring PCI with DES implantation; target-vessel reference size of 2.5 to 4.5 mm	Inability to provide informed consent; participation in another study that has not reached the primary endpoint; contra-indication for DAPT, heparin, contrast agent, or DES components; planned surgery within three months; life expectancy of <1 year; female of childbearing potential; lesion amendable for DEB.
Romaguera et al. (2016) [29]	Multi-RCT	PF-SES	PP-EES	Diabetic patients with silent ischemia, stable angina, or non-ST-segment elevation myocardial infarction have a de novo lesion per coronary artery of 12-25 mm and a reference diameter of 2.5-3.5 mm, treated with a single stent.	ST-segment elevation myocardial infarction left main or ostial left descending artery stenosis, bifurcations, stent restenosis, chronic renal failure, glomerular filtration rate >30 ml/min, left ventricular ejection fraction <30%, DM treated with diet and lifestyle.
Shiratori et al. (2014) [30]	Multi-RCT	PF-PES	PP-PES	Stable or unstable angina or non-ST-elevation myocardial infarction, age ≥18, native and de novo lesions, reference vessel diameters.	Acute coronary syndrome, renal insufficiency, liver failure, and other pathologies that can reduce life expectancy.
Jensen et al. (2018) [31]	Multi-RCT	PF-BES	BP-SES	Chronic coronary artery disease or acute coronary syndromes.	Life expectancy less than one year, allergy to aspirin, clopidogrel, ticagrelor, sirolimus, or biolimus, Participation in another randomized trial, Unable to provide written informed consent
Zhang et al. (2013) [32]	Single-RCT	PF-PES	PP and BP- SES	Patients older than 18 years, stable angina or unstable angina syndromes, acute MI patients needing emergency PCI, and patients with the same type of randomly assigned stent in multiple lesions and in a single lesion needing two or more stents.	Patients with a target lesion in the left main artery, multiple stent types, or an allergy to study medication, patients more likely to require multiple stents.
Natsuaki et al. (2013) [33]	Multi-RCT	PF-BES	PP-EES	Patients scheduled for percutaneous coronary intervention (PCI) using DES	None
Zhang et al. (2014) [34]	Multi-RCT	PF-SES	PP-SES	Age ≥18 with two de novo native coronary artery lesions, target lesion with stenosis ≥70%, visual reference vessel diameter ≤2.5-4.0 mm, and visual lesion length ≤40 mm.	Acute myocardial infarction with left main coronary artery disease, true bifurcation lesion with side branch diameter ≥2.25 mm, chronic total.
Windecker et al. (2020) [35]	Multi-RCT	PF-UES	PP-ZES	Age ≥75 years, any prior documented intracerebral bleed, stroke, hospital admission, non-skin cancer, renal failure, thrombocytopenia, severe chronic liver disease, variceal hemorrhage, ascites, hepatic encephalopathy or jaundice.	Active bleeding, cardiogenic shock, hypersensitivity to aspirin, heparin, and bivalirudin, P2Y12 inhibitors, mTOR inhibitors, cobalt, nickel, platinum, iridium, chromium, molybdenum, polymer coatings, stainless steel, zinc, or sensitivity to contrast agents, pregnant and nursing women, planned PCI procedure after one month of the index procedure. Participation in another clinical research within 12 months of the index surgery, PCI during the previous six months for a lesion different than the index procedure's target lesion, and a life expectancy of less than two years.
Ellert-Gregersen et al. (2022) [36]	Multi-RCT	PF-BES	BP-SES	Eighteen years old with chronic stable coronary artery disease or acute coronary syndrome and at least one coronary lesion with >50% diameter stenosis, requiring treatment with a DES. If multiple lesions were treated, the allocated study stent must be used in all lesions.	Life expectancy of <1 year; an allergy to aspirin, clopidogrel, ticagrelor, prasugrel, sirolimus, or biolimus; participation in another randomized stent trial; or an inability to provide written informed consent.
van Hemert et al. (2021) [37]	Multi-RCT	PF-AES	PP-ZES	Clinical symptoms of ischemia indicating the presence of coronary artery stenosis requiring PCI	-
Rozemeijer et al. (2019) [38]	Prospective registry	PF‐AES	PP-ZES	Stable coronary artery disease or acute coronary syndromes, and at least one coronary artery lesion with more than 50% diameter stenosis eligible for treatment with either PF‐AES or PP‐ZES implantation between January 2014 and February 2016	implantation of bare‐metal stents, a combination of DES, or revascularization prior to transcatheter aortic valve implantation.
Gallone et al. (2021) [39]	Multicenter observational	PF-BES	PP-ZES	Chronic coronary artery disease or acute coronary syndromes.	Patients with a target lesion in the left main artery, multiple stent types, or an allergy to study medication, patients more likely to require multiple stents.
Koch et al. (2021) [40]	Multi-RCT	PF-sirolimus- and probucol-eluting stent	PP-ZES	Ischemic symptoms or evidence of myocardial ischemia; 50% de novo stenosis in native coronary vessel.	Patients with a life expectancy <12 months, allergy to study medications, pregnancy or positive pregnancy test.
Loewenstein et al. (2022) [41]	Prospective, open-label registry	PF	PP	All comers	-
